# Editorial Correspondence

**Published:** 1877-10

**Authors:** Robt. B. Trezevant

**Affiliations:** Des arc, Arkansas


Des Arc, Arkansas, Sept. 29, 1877.

*My Dear Doctor :*

I noticed an article in the August number of your Journal severely condemning the use of morphine hypodermically. I presume that it astonished many of the profession, as it did myself. The writer (Dr. Ingals) gives a few cases in which the consequences of the practice were disastrous, without comparing them with the thousands of cases in which the hypodermic use of morphine proves both harmless and highly beneficial. I have seen identically the same bad effect as Dr. Ingals describes from the use of this drug given by the stomach to persons who, from an idiosyncracy, could not take morphine at all. I could cite as many of these as Dr. I. can ; yet I would not, on this account, condemn the use of morphine by stomach. My own experience teaches me that any one who can bear the use of morphine at all, will do so as well hypodermically as otherwise.

Your obedient servant,

Robt. B. Trezevant, M.D.

We heartily endorse the above views of our correspondent, and intended, in the number of our Journal which contained the selected article referred to above, to have mentioned some other facts which may account for the erroneous impression that the hypodermic use of opium is more dangerous than other manner of administration. Three facts must be remembered in the investigation of this question: 1st, that, as our correspondent has stated, some persons, from idiosyncrasy, do not tolerate opium in any form of preparation or manner of administration ; 2d, that too large a dose of this remedy may produce unpleasant symptoms, particularly in certain forms of disease; and 3d, that less quantity is required to produce a given effect, when hypodermically administered, than when given in the stomach. The absorption is more rapid from the cellular tissue beneath the skin than from the mucous membrane of the stomach, even when this viscus is empty, and much more so when the remedy is mixed up with solid or fluid ingesta, so that only a small portion is at any time in contact with the absorbing surface. Moreover, when thus slowly absorbed the full effect of the remedy is never experienced, for the action of the first absorbed portion will have been exhausted before the last has reached the brain, or even the blood vessels.—[Ed.]

---

